# Vitamin B_12_ Status in Pregnant Adolescents and Their Infants

**DOI:** 10.3390/nu11020397

**Published:** 2019-02-13

**Authors:** Julia L. Finkelstein, Ronnie Guillet, Eva K. Pressman, Amy Fothergill, Heather M. Guetterman, Tera R. Kent, Kimberly O. O’Brien

**Affiliations:** 1 Division of Nutritional Sciences, Cornell University, Ithaca, NY 14853, USA; af544@cornell.edu (A.F.); hg384@cornell.edu (H.M.G.); trk8@cornell.edu (T.R.K.); koo4@cornell.edu (K.O.O.); 2 University of Rochester Medical Center, Rochester, NY 14642, USA; ronnie_guillet@urmc.rochester.edu (R.G.); eva_Pressman@urmc.rochester.edu (E.K.P.)

**Keywords:** vitamin B_12_, micronutrients, pregnancy, adolescents, folate

## Abstract

Vitamin B_12_ deficiency has been associated with increased risk of adverse pregnancy outcomes. Few prospective studies have investigated the burden or determinants of vitamin B_12_ deficiency early in life, particularly among pregnant adolescents and their children. The objectives of this study were to determine the prevalence of vitamin B_12_ deficiency and to examine associations between maternal and neonatal vitamin B_12_ status in a cohort study of healthy pregnant adolescents. Serum vitamin B_12_ and folate concentrations were measured in adolescents at mid-gestation (*n* = 124; 26.4 ± 3.5 weeks) and delivery (*n* = 131; 40.0 ± 1.3 weeks), and in neonates at birth using cord blood. Linear regression was used to examine associations between maternal and neonatal vitamin B_12_ status. Although the prevalence of vitamin B_12_ deficiency (<148.0 pmol/L; 1.6%) in adolescents was low during pregnancy, 22.6% of adolescents were vitamin B_12_ insufficient (<221.0 pmol/L; 22.6%) at mid-gestation. Maternal vitamin B_12_ concentrations significantly decreased from mid-gestation to delivery (*p* < 0.0001), and 53.4% had insufficient vitamin B_12_ status at delivery. Maternal vitamin B_12_ concentrations (*p* < 0.001) and vitamin B_12_ deficiency (*p* = 0.002) at delivery were significantly associated with infant vitamin B_12_ concentrations in multivariate analyses, adjusting for gestational age, maternal age, parity, smoking status, relationship status, prenatal supplement use, pre-pregnancy body mass index, race, and intake of vitamin B_12_ and folate. Maternal vitamin B_12_ concentrations significantly decreased during pregnancy and predicted neonatal vitamin B_12_ status in a cohort of healthy pregnant adolescents.

## 1. Introduction

Vitamin B_12_ deficiency (serum vitamin B_12_ <148.0 pmol/L) is a major public health problem globally [1,2]. Although the overall prevalence of vitamin B_12_ deficiency in the United States is estimated to be relatively low (6%), the burden of vitamin B_12_ deficiency is higher in the elderly, pregnant women, and young children (6–25%) [3]. Pregnant adolescents are at increased risk for a variety of micronutrient deficiencies and pregnancy complications, though there is limited data from this high-risk obstetric population. 

Vitamin B_12_ deficiency in pregnancy has been associated with increased risk of pregnancy outcomes, including spontaneous abortion, pregnancy loss, intrauterine growth restriction, low birthweight (<2500 g), and neural tube defects (NTDs) [4,5,6,7,8,9,10,11,12,13,14,15]. Inadequate supply of vitamin B_12_ in pregnancy and early childhood can lead to long-term deficits in growth development in children [16,17].

Maternal vitamin B_12_ concentrations during pregnancy are thought to predict fetal [18,19,20,21,22,23,24,25,26] and early infant [25,27,28,29] vitamin B_12_ status. Previous cross-sectional studies in Norway, Turkey, Germany, United Kingdom, Serbia, and Brazil have noted a significant correlation between maternal and infant vitamin B_12_ status at delivery [18,20,21,22,30,31,32,33]; however, in one study in Belgium, maternal and infant vitamin B_12_ concentrations were not significantly correlated [34]. In one study in Germany, maternal serum vitamin B_12_ and holotranscobalamin (holoTC) concentrations at delivery were significantly correlated with cord blood holoTC concentrations (*p* < 0.05) [18]. In contrast, findings from cross-sectional studies examining the associations between maternal and infant vitamin B_12_ concentrations later in the postpartum period have been heterogeneous [35,36,37,38,39,40]. Maternal vitamin B_12_ and holoTC concentrations were significantly correlated with infant vitamin B_12_ concentrations in the first month (i.e., 2–30 days) postpartum in a study in Turkey [38]. In analyses in mother–infant dyads in the first 6 months postpartum, maternal and infant vitamin B_12_ concentrations were significantly associated in Canada and Cambodia (i.e., 3–27 weeks) [37], but not in India (i.e., 1–6 months) [35]. 

Prospective studies to date in The Netherlands, Norway, Turkey, India, and Spain have reported significant associations [19,23,24,26,29] between maternal vitamin B_12_ status during pregnancy and infant vitamin B_12_ status in cord blood or serum. In a prospective study in India, maternal vitamin B_12_ status during pregnancy was associated with infant vitamin B_12_ concentrations at 6 weeks of age [28]. In contrast, a study in Norway was conducted to examine the associations between maternal vitamin B_12_ biomarkers during pregnancy and vitamin B_12_ status in infants at birth and 6 months of age; maternal vitamin B_12_ concentrations did not significantly predict cord blood or infant vitamin B_12_ status, although there were significant associations noted for other biomarkers (i.e., maternal holoTC, holohaptocorrin (holoHC), and methylmalonic acid (MMA)) [25]. Although some studies to date have been conducted to examine vitamin B_12_ status in pregnant adolescents [41,42,43], most studies investigating the associations between maternal and infant vitamin B_12_ status have been conducted among adult pregnant women (i.e., 18 to 40 years). Of these studies, three cross-sectional studies reported participants which included adolescents, with age ranges of 15 to 38 years [32], 16 to 40 years [38], and 17 to 43 years [27]. However, adolescents comprised a small proportion (<15%) of the sample, and data presented were not stratified by age group, which constrained analysis and interpretation of findings for adolescents. There are limited prospective studies on the associations between maternal and infant vitamin B_12_ status conducted in high-risk obstetric groups such as adolescents.

Pregnant adolescents are at increased risk for a variety of micronutrient deficiencies and pregnancy complications [41,44]. The inadequate dietary intake of key nutrients among adolescents in industrialized countries [45], coupled with increased nutritional requirements for growth and development, warrants concern for health outcomes among pregnant adolescents. However, few data exist on the extent of vitamin B_12_ deficiency or its implications for fetal and child health in this high-risk obstetric population, which comprises over 5% of the US population and 11% globally [46,47]. Well-designed prospective studies are needed to elucidate the burden of vitamin B_12_ insufficiency in this key high-risk population and its implications for maternal and child health. 

We, therefore, conducted a prospective observational analysis to: (1) determine the prevalence of vitamin B_12_ deficiency and insufficiency in pregnant adolescents and their infants; and (2) examine the associations of maternal and neonatal vitamin B_12_ status in healthy pregnant adolescents.

## 2. Materials and Methods 

### 2.1. Study Population

Participants included in this study were enrolled in one of two prospective cohort studies funded by the United States Department of Agriculture (USDA). One study examined maternal and fetal bone health among pregnant adolescents (“bone health study”) and collected maternal blood samples at mid-gestation and delivery, and cord blood samples at delivery. The other study evaluated iron status and anemia through gestation in pregnant adolescents aged 13 to 18 years and their infants (“anemia study”), and collected maternal and cord blood samples only at delivery. Both studies were observational cohort studies and not clinical trials (and, thus, do not need to be registered, as per protocol for clinical trials). Pregnant adolescents were recruited between 2006 and 2012, from the Rochester Adolescent Maternity Program (RAMP) in Rochester, New York. 

Adolescents were eligible to participate if their pregnancies were 12 to 30 weeks in gestation at the time of the adolescents’ enrollment in prenatal care at RAMP, and if the adolescents were healthy and carrying a single fetus. Adolescents were excluded if they had any known medical complications, including diabetes, preeclampsia, gestational hypertension, eating disorders, gastrointestinal diseases, HIV infection, or any other diagnosed medical conditions. Data on maternal and neonatal iron status [48,49] and on vitamin B_12_ transporters in placental tissue from this population [50] have been previously reported.

### 2.2. Ethics

Written informed consent was obtained from all study participants. The research protocol and study procedures were approved by the Institutional Review Boards (IRB) at Cornell University and the University of Rochester. The IRB approval included laboratory analyses of micronutrients in maternal and infant cord blood samples, including vitamin B_12_ and folate concentrations. 

### 2.3. Follow-Up Procedures

**Figure 1:**
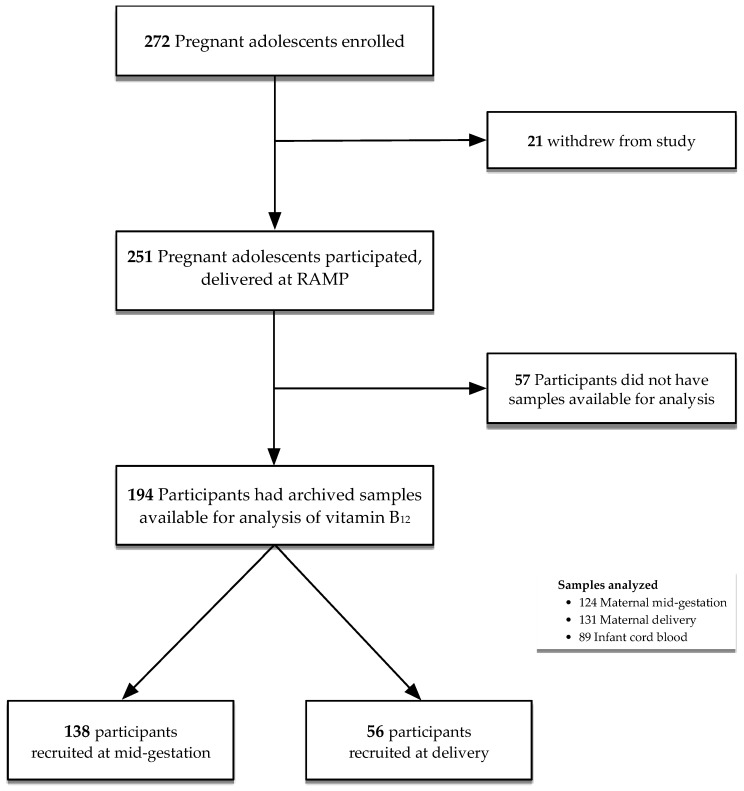
Participant flow diagram.

Structured interviews were conducted to collect demographic information, including maternal age, educational level, socioeconomic status, and obstetric history at the baseline clinic visit. Detailed clinical, dietary (i.e., 24-h dietary recall), anthropometric, and biochemical data were collected at each visit. The participant recruitment and flow chart are presented in Figure 1. Of 251 participants who delivered at RAMP, a total of 194 participants (*n* = 138 participants in the bone study, recruited at mid-gestation; *n* = 56 participants in the anemia study, recruited at delivery) had archived blood samples available for analysis (Figure 1). All adolescents attending the Rochester Adolescent Maternity Program were prescribed a prenatal supplement as standard of care, which contained 27 mg iron, 12 μg vitamin B_12_, 1000 μg folic acid, and other micronutrients (i.e., 1200 μg vitamin A, 120 mg vitamin C, 10 μg vitamin D_3_, 22 mg vitamin E, 1.84 mg thiamin, 3 mg riboflavin, 20 mg niacin, 10 mg vitamin B_6_, 200 mg calcium, 25 mg zinc, and 2000 μg copper).

### 2.4. Laboratory Analyses

Non-fasting maternal venous blood samples (mid-gestation, delivery) and infant cord blood samples were allowed to clot at room temperature, separated by centrifugation, processed, and stored below −80 °C until analysis. A total of 124 maternal mid-gestation (26.4 ± 3.5 weeks), 131 maternal delivery (40.0 ± 1.3 weeks), and 89 infant cord blood samples were available for analysis. 

Vitamin B_12_ concentrations were measured by electrochemiluminescence using the IMMULITE 2000 immunoassay system (Siemens Medical Solutions Diagnostics, Los Angeles, CA, USA). Three levels of controls (Bio-Rad) were used for serum vitamin B_12_, with inter-assay coefficients of variation (CV) of 4.2% for Level 1 and 4.8% for Level 3. Serum folate concentrations were measured using the IMMULITE 2000 immunoassay system. The Bio-Rad Liquichek Immunoassay Plus Control (High & Low) were used as controls, with intra-assay precision of 6.7% and inter-assay precision of 6.6%.

### 2.5. Definitions of Outcomes 

Conventional cutoffs were used to categorize variables where available; otherwise, medians of variables were defined based on their distributions in the population. Vitamin B_12_ deficiency and insufficiency were defined, following standard Centers for Disease Control and Prevention (CDC) definitions, as less than 148 pmol/L and less than 221.0 pmol/L, respectively [51]. Anemia was defined as hemoglobin <11.0 g/dL during the first and third trimesters, <10.5 g/dL during the second trimester, and <11.0 g/dL at delivery; and anemia status was adjusted for race [52]. Folate deficiency was defined as <6.8 nmol/L [51]. Maternal BMI was defined as the ratio of weight in kg to height in m^2^ (kg/m^2^), and categorized as <18.5, 18.5 to <25.0, 25.0 to <30.0, and ≥30.0 kg/m^2^, in accordance with the CDC and World Health Organization (WHO) classifications [53]. Infant low birthweight was defined as <2500 g. Infant ponderal index was calculated as the ratio of weight in g to length in cm^3^ (g/cm^3^ × 100).

### 2.6. Statistical Analyses 

Binomial and linear regression models were used to examine the associations of maternal vitamin B_12_ status at mid-gestation and delivery with infant vitamin B_12_ status at birth. Binomial regression models were used to obtain risk ratio (RR) estimates for dichotomous variables [54,55,56]. Non-normally distributed variables were natural logarithmically transformed to ensure normality before further analysis. We also examined the associations between maternal and infant folate status. The values in Table 1 are presented as non-transformed values for interpretation purposes. 

We explored potential nonlinearity of the relationships between covariates and outcomes nonparametrically, using stepwise restricted cubic splines [57,58]. If nonlinear associations were not reported, they were not significant. The Rothman and Greenland approach was used to evaluate and adjust for confounding, in which all known or suspected risk factors for the outcome which lead to a >10% change-in-estimate were included in the models [59]. Observations with missing data for covariates were retained in analyses using the missing indicator method [60]. Statistical analyses were conducted using SAS software, version 9.4 (SAS Institute, Inc., Cary, NC, USA).

## 3. Results

### 3.1. Baseline Characteristics

The characteristics of participants in this study are presented in Table 1. Participants and their infants enrolled in the overall cohort studies and in the current study (i.e., with available serum vitamin B_12_ data) were similar in terms of baseline characteristics, including maternal age, socioeconomic characteristics, and nutritional status. A total of 194 participants had archived samples available for analysis; 138 of these participants were recruited at mid-gestation (bone health study), and 56 participants were recruited at delivery (anemia study) (Figure 1). We also examined potential differences in demographic, socioeconomic, and nutritional factors between participants in the two cohort studies. These variables were identified *a priori* as potential confounders and were considered and adjusted for in all of the multivariate analyses. Vitamin B_12_ and folate concentrations were analyzed in maternal samples that were collected at mid-gestation (*n* = 124) and delivery (*n* = 131); and in infant cord blood samples (*n* = 89). 

### 3.2. Maternal and Neonatal Vitamin B_12_ Status

Maternal and neonatal vitamin B_12_ status are presented in Table 2. At the mid-gestation visit (*n* = 124; 26.4 ± 3.5 weeks gestation), 1.6% of women were vitamin B_12_ deficient (*n* = 2/124; <148.0 pmol/L), and 22.6% were vitamin B_12_ insufficient (*n* = 28/124; <221.0 pmol/L). Maternal serum vitamin B_12_ concentrations significantly decreased from mid-gestation to delivery (*n* = 61; 39.9 ± 1.0 weeks; mid-gestation: median = 358.9, interquartile range (IQR) = 233.9, 400.7 vs. delivery: median = 226.2, IQR = 185.2, 311.8; *p* < 0.0001). 

The prevalence of maternal vitamin B_12_ insufficiency at delivery (*n* = 70/131; 53.4%) was significantly higher than at mid-gestation (*n* = 28/124; 22.6%, *p* < 0.05). The prevalence of vitamin B_12_ insufficiency was low in infants at birth: 0.0% were vitamin B_12_ deficient (<148.0 pmol/L), and 2.3% were vitamin B_12_ insufficient (<221.0 pmol/L). No mothers or infants were folate deficient (<6.8 nmol/L) or insufficient (<10.0 nmol/L) during this study. 

The associations between maternal and infant serum vitamin B_12_ concentrations are presented in Table 3. Maternal vitamin B_12_ status at mid-gestation was not significantly associated with infant serum vitamin B_12_ concentrations (*p* > 0.05).

At delivery, maternal serum vitamin B_12_ concentrations (*p* < 0.001) and vitamin B_12_ deficiency (*p* < 0.0001) were significantly associated with infant serum vitamin B_12_ concentrations in multivariate analyses, adjusting for gestational age at sample collection, maternal age, parity, smoking status, relationship status, reported prenatal supplement use, pre-pregnancy BMI, race, and intake of vitamin B_12_ and folate. Similarly, maternal vitamin B_12_ insufficiency at delivery was significantly associated with infant serum vitamin B_12_ concentrations (*p* < 0.01) in multivariate analyses, adjusting for gestational age at sample collection, maternal age, parity, smoking status, relationship status, reported prenatal supplement use, pre-pregnancy BMI, race, and intake of vitamin B_12_ and folate. Maternal serum folate concentrations were not significantly associated with infant serum vitamin B_12_ concentrations (*p* > 0.05).

The associations between maternal vitamin B_12_ and folate statuses and infant serum folate concentrations are presented in Table 4. Maternal serum folate concentrations at mid-gestation were not significantly associated with infant serum folate concentrations (*p* > 0.05).

Maternal serum folate concentrations at delivery were significantly associated with infant serum folate concentrations (*p* < 0.0001) in multivariate analyses, adjusting for gestational age at sample collection, maternal age, parity, smoking status, relationship status, prenatal supplement use, pre-pregnancy BMI, race, and intake of vitamin B_12_ and folate. Similarly, lower maternal serum folate concentrations (<40.0 nmol/L) at delivery were associated with lower infant serum folate concentrations (*p* < 0.0001) in multivariate analyses, adjusting for gestational age at sample collection, maternal age, parity, smoking status, relationship status, prenatal supplement use, pre-pregnancy BMI, race, and intake of vitamin B_12_ and folate.

## 4. Discussion

In this prospective analysis among pregnant adolescents, maternal vitamin B_12_ concentrations significantly decreased during pregnancy and predicted neonatal vitamin B_12_ status. Although the prevalence of vitamin B_12_ deficiency (<148.0 pmol/L; 1.6%) was low in adolescents during pregnancy, 22.6% of adolescents were vitamin B_12_ insufficient (<221.0 pmol/L; 22.6%) at mid-gestation. Maternal serum vitamin B_12_ concentrations decreased significantly during pregnancy, and at delivery, 15.3% of mothers were vitamin B_12_ deficient and 53.4% were vitamin B_12_ insufficient (Table 2).

This is among the first studies conducted to date to examine the burden of vitamin B_12_ deficiency in pregnant adolescents and its association with neonatal vitamin B_12_ status in this high-risk obstetric population. The prevalence of vitamin B_12_ deficiency in this study was low (1.6% mid-gestation, 15.3% delivery) and similar to a previous study conducted in Spain among pregnant adolescents (vitamin B_12_ deficiency, T1: 8.3%) [42]. However, the prevalence of vitamin B_12_ deficiency noted in this study was lower than previous studies conducted in pregnant adolescents in Canada (median, T3: 158 pmol/L, IQR: 114, 207 pmol/L; vitamin B_12_ <148.0 pmol/L: 43%) [43] and in Venezuela (vitamin B_12_ <200.0 pg/mL (<148.0 pmol/L), T1: 50.0%, T2: 58.8%, T3: 72.5%) [61]. Maternal vitamin B_12_ concentrations in our study were also higher than in a previous study in pregnant adolescents in the United Kingdom (geometric mean, Trimester 3 (T3): 177 pmol/L, 95% CI: 169, 185 pmol/L) [41].

The prevalence of vitamin B_12_ insufficiency (<221.0 pmol/L), however, was high in this study at both mid-gestation (22.6%) and delivery (53.4%). Although all participants were prescribed prenatal vitamins containing vitamin B_12_ and folic acid, self-reported adherence to prenatal supplements was low. Additionally, while most participants reported dietary intake of vitamin B_12_ at or above the RDA for this group (i.e., median (IQR): 4.5 (2.6, 6.6) µg/day vs. RDA: 2.6 µg/day), approximately 25% of participants reported dietary intake below the RDA. In addition to low dietary intake of vitamin B_12_, vitamin B_12_ absorption could also be impaired by inadequate bioavailability, losses from processing and cooking animal-source foods, high dose folic acid, metabolic changes during pregnancy (e.g., hemodilution, fetal transfer), gastrointestinal symptoms, infections, and medications [4,62]. For example, since vitamin B_12_ is bound to protein carriers in the food matrix, vitamin B_12_ bioavailability may vary by food source [62,63].

The decline in maternal vitamin B_12_ concentrations during gestation in this study is also consistent with previous studies in adult pregnant women in Canada [64], Spain [26], Norway [25,29], and India [28,65], and in 12 of 13 longitudinal studies included in a systematic review of vitamin B_12_ status and birthweight in adult pregnant women [66]. The observed decrease in vitamin B_12_ concentrations throughout pregnancy could be due to hemodilution, increased protein synthesis, increased requirements for methyl donors during gestation, or a low intake or adherence to prenatal supplements to meet increased requirements [67]. However, there are limited data from pregnant adolescents, who have higher nutritional requirements for their own growth. 

The prevalence of vitamin B_12_ deficiency and insufficiency in infants was low in this study (0–3%). Infant vitamin B_12_ concentrations were 2.5-fold higher than maternal vitamin B_12_ concentrations at delivery. These findings are consistent with previous studies in adult pregnant women, which have reported neonatal vitamin B_12_ concentrations 27% to 100% higher than maternal concentrations at delivery [18,19,21,26,30,31,33] and mid-gestation [23], although this has not been reported in all studies [22,25,28,29]. Higher vitamin B_12_ concentrations in offspring indicate active transfer to the fetus, which may occur due to upregulation of placental B_12_ transporter proteins or other active transport mechanisms that have yet to be established.

In this study, maternal vitamin B_12_ status at delivery, but not at mid-gestation, was significantly associated with infant vitamin B_12_ status. Maternal vitamin B_12_ status at delivery has been associated with vitamin B_12_ status in offspring at birth in previous cross-sectional studies [18,20,21,22,30,31,32,33]. There are, however, limited prospective data on maternal vitamin B_12_ status during pregnancy and its association with infant vitamin B_12_ status—particularly among adolescents—to compare findings. Evidence from studies in adult pregnant women have reported significant correlations between maternal vitamin B_12_ status during pregnancy and their infants [19,23,24,26,29]. Few prospective analyses to date have considered potential confounders of these associations in multivariate analyses [25,28]. In a recent study in adult pregnant women (median age = 22, IQR = 20–24 years) in Southern India, maternal vitamin B_12_ status during each trimester was associated with infant vitamin B_12_ status at 6 weeks of age [28], even after adjusting for maternal vitamin B_12_ supplementation. Similarly, a study conducted among pregnant women (mean age = 29.9, SD = 4.4 years) in Norway found that maternal vitamin B_12_ levels did not significantly predict cord blood or infant vitamin B_12_ status, although other vitamin B_12_ biomarkers (i.e., maternal holoTC, holoHC, MMA) were associated [25]. 

This study has several limitations. Neonatal micronutrient status was assessed at a single time point from cord blood, precluding our ability to evaluate longer-term impacts on infant vitamin B_12_ status or functional outcomes. Longitudinal data on maternal vitamin B_12_ concentrations were available only from a subset of participants in the parent cohort studies, limiting our ability to examine changes in vitamin B_12_ concentrations during pregnancy. Although participants in both cohort studies had similar sociodemographic characteristics (e.g., maternal age, gestational age at initiation of prenatal care, adherence to prenatal vitamins, gestational age at delivery), participants enrolled at mid-gestation (bone study) were more likely to be participants in the Special Supplemental Nutrition Program for Women, Infants, and Children (WIC) program, current smokers, Caucasian, primiparous, and had higher self-reported dietary intake of vitamin B_12_ and folate, compared to participants who were recruited at delivery (anemia). All of these variables were identified *a priori* as potential confounders and were considered and adjusted for in multivariate analyses; however, there may be residual confounding due to additional factors that were not evaluated or adjusted for in these studies. Vitamin B_12_ concentrations assessed at mid-gestation may not reflect vitamin B_12_ status during the relevant etiologic period periconceptionally or for maternal–fetal transfer of cobalamin and subsequent infant status and perinatal outcomes [68]. Additionally, serum folate is a biomarker of short-term dietary intake and does not reflect longer-term or usual intake. Vitamin B_12_ and folate assessments were also based on a single biomarker (i.e., total serum vitamin B_12_ and serum folate concentrations). Inclusion of additional circulating (i.e., holo-transcobalamin) and functional (i.e., methylmalonic acid) biomarkers of vitamin B_12_ metabolism and erythrocyte folate concentrations would improve assessment and interpretation of findings in mother–infant dyads [4]. Additionally, while the low prevalence of vitamin B_12_ deficiency in this study is similar to previous research in pregnant adolescents in Canada and the United Kingdom, a study population of generally adequate vitamin B_12_ status limits the generalizability of results to other populations that may be at greater risk for vitamin B_12_ deficiency, particularly in resource-limited settings [41,43]. Findings should also be interpreted in the context of a folate-replete population (i.e., among participants prescribed high-dose prenatal folic acid (1000 μg) and in a population exposed to folic acid fortification); this also limits the generalizability of findings to other settings. Finally, although findings from this study demonstrated an association of maternal and infant vitamin B_12_ status at delivery, the interpretation of these findings is not causal. Future prospective studies are needed to examine mechanisms of vitamin B_12_ transfer to the fetus and to determine the impact of vitamin B_12_ status on maternal and child health outcomes.

## 5. Conclusions

In summary, in this cohort of healthy pregnant adolescents, maternal vitamin B_12_ concentrations significantly decreased during pregnancy and predicted infant vitamin B_12_ status. This is one of the first prospective studies to date to evaluate the burden of vitamin B_12_ insufficiency in pregnant adolescents and their infants, a population that is at high risk for both micronutrient deficiencies and pregnancy complications. Findings suggest that vitamin B_12_ deficiency is an important public health problem in this high-risk obstetric population. Future research is needed to increase vitamin B_12_ status and improve the health of adolescent mothers and their children. 

## Figures and Tables

**Table 1:** Characteristics of the study population.

Variables ^a^	Original Cohort (*n* = 251)	Current Study (*n* = 194)	Recruited at Mid-Gestation (*n* = 138)	Recruited at Delivery (*n* = 56)
**Maternal**				
Age at enrollment, years	17.3 (16.5, 18.1)	17.3 (16.5, 18.1)	17.3 (16.4, 18.1)	17.3 (16.6, 18.1)
Age at delivery, years	17.5 (16.7, 18.3)	17.6 (16.8, 18.4)	17.6 (16.7, 18.4)	17.4 (16.9, 18.2)
<16 years, % (*n*)	12.0 (30)	9.8 (19)	9.4 (13)	10.7 (6)
Gestational age at delivery, weeks	39.9 (38.7, 40.7)	40 (39.0, 40.9)	40.0 (38.9, 40.9)	40.0 (39.2, 41.0)
Pre-term (<37 weeks), % (*n*)	8.0 (20)	7.8 (15)	8.8 (12)	5.4 (3)
Parity ≥1, % (*n*)	17.3 (43)	15.1 (29)	8.7 (12)	30.9 (17)
Smoking at enrollment, % (*n*)				
Never a smoker	77.8 (189)	78.5 (150)	77.5 (107)	81.1 (43)
Past smoker	15.2 (37)	14.4 (27)	12.3 (17)	18.9 (10)
Current smoker	7.0 (17)	7.3 (14)	10.1 (14)	0.0 (0)
Relationship status ^b^, % (*n*)	13.5 (33)	10.5 (20)	1.5 (2)	34.0 (18)
WIC ^c^ program participant	60.9 (148)	63.2 (120)	80.0 (100)	37.7 (20)
Self-reported prenatal supplement use, % (*n*)				
≥2 pills per week	54.1 (131)	55.5 (106)	56.6 (77)	52.7 (29)
Dietary folate, µg/day	617.2 (397.0, 948.9)	617.2 (400.8, 950.45)	692.7 (464.2, 1020.6)	415.3 (283.9, 624.7)
Dietary vitamin B_12_, µg/day	4.6 (2.7, 6.5)	4.6 (2.7, 6.6)	5.0 (3.7, 6.9)	2.8 (1.4, 5.2)
Pre-pregnancy BMI, kg/m^2^	23.5 (20.8, 28.0)	23.7 (20.8, 28.0)	23.3 (20.8, 28.1)	24.7 (20.8, 27.9)
<18.5 kg/m^2^, % (*n*)	6.9 (17)	7.3 (14)	6.62 (9)	9.1 (5)
≥18.5 to <25 kg/m^2^, % (*n*)	54.3 (133)	52.4 (100)	55.2 (75)	45.5 (25)
≥25.0 to <30 kg/m^2^, % (*n*)	20.8 (51)	21.5 (41)	19.9 (27)	25.5 (14)
≥30 kg/m^2^, % (*n*)	18.0 (44)	18.9 (36)	18.4 (25)	20.0 (11)
Gestational weight gain (GWG), kg	15.9 (11.8, 20.5)	16.4 (11.8, 20.5)	15.5 (11.8, 20.5)	17.3 (12.3, 21.4)
Inadequate ^d^ GWG, % (*n*)	15.0 (36)	13.9 (26)	14.3 (19)	13.0 (7)
Within IOM range, % (*n*)	22.9 (55)	24.0 (45)	26.3 (35)	18.5 (10)
Excessive GWG, % (*n*)	62.1 (149)	62.0 (116)	59.0 (79)	68.5 (37)
Race, % (*n*)				
Caucasian	27.9 (70)	29.4 (57)	33.3 (36)	19.6 (11)
African American	71.3 (179)	69.6 (135)	65.2 (90)	80.4 (45)
Native American	0.8 (2)	1.0 (2)	1.5 (2)	0.0 (0)
Ethnicity, % (*n*)				
Hispanic	24.3 (61)	26.3 (51)	24.6 (34)	30.4 (17)
**Infant**				
Birthweight, g	3206.0 (2904.0, 3550.0)	3266.0 (2928.0, 3581.0)	3258.0 (2892.0, 3581.0)	3318.5 (3055.5, 3584.0)
Birth length, cm	51.0 (49.0, 52.7)	51.3 (49.5, 52.9)	51.0 (49.5, 52.5)	52.0 (50.0, 53.5)
Weight-for-length *z*-score < −2, % (*n*)	27.0 (60)	27.0 (47)	27.3 (35)	26.1 (12)
Ponderal index, g/cm^3^ × 100	2.4 (2.2, 2.7)	2.4 (2.3, 2.7)	2.4 (2.2, 2.7)	2.4 (2.3, 2.6)
Male sex, % (*n*)	52.8 (132)	51.0 (99)	50.7 (70)	51.8 (29)

^a^ Values are median interquartile range (IQR) and % (*n*); ^b^ Data presented are adolescents that report being in a relationship during pregnancy vs. single; ^c^ The Special Supplemental Nutrition Program for Women, Infants, and Children (WIC); ^d^ Gestational Weight Gain: categorized as inadequate or excessive, using Institute of Medicine (IOM) recommendations that vary based on pre-partum body mass index (BMI).

**Table 2:** Maternal and infant vitamin B_12_ and folate status.

	Maternal				Infant		
	Mid-Gestation		Delivery		Cord Blood		
Variables ^a^	Total	Total	Recruited at Mid-Gestation	Recruited at Delivery	Total	Mothers Recruited at Mid-Gestation	Mothers Recruited at Delivery
*n*	124	131	75	56	89	58	31
Serum vitamin B_12_, pmol/L	343.7 (237.8, 400.7)	216.2 (161.6, 297.8)	216.2 (173.4, 311.8)	211.2 (158.7, 267.0)	597.0 (471.6, 796.3)	569.4 (478.6, 844.3)	602.9 (406.6, 722.1)
<148.0 pmol/L	1.6 (2)	15.3 (20)	14.7 (11)	16.1 (9)	0.0 (0)	0.0 (0)	0.0 (0)
≥148 to <221.0 pmol/L	21.0 (26)	38.2 (50)	37.3 (28)	39.3 (22)	2.3 (2)	0.0 (0)	6.5 (2)
≥221 pmol/L	77.4 (96)	46.6 (61)	48.0 (36)	44.6 (25)	99.8 (87)	100.0 (58)	93.5 (29)
*n*	122	130	74	56	86	55	31
Serum folate, nmol/L	39.3 (31.7, 50.5)	39.7 (31.8, 50.4)	42.8 (32.2, 51.4)	37.7 (28.8, 48.4)	66.7 (53.1, 85.5)	66.3 (52.1, 84.4)	67.7 (55.5, 98.4)
≤29.45^b^ nmol/L	19.7 (24)	20.0 (26)	13.5 (10)	28.6 (16)	2.3 (2)	3.6 (2)	0.0 (0)
>29.45, ≤35.79 nmol/L	20.5 (25)	16.9 (22)	18.9 (14)	14.3 (8)	2.3 (2)	3.6 (2)	0.0 (0)
>35.79, ≤43.94 nmol/L	19.7 (24)	20.0 (26)	17.6 (13)	23.2 (13)	4.7 (4)	5.5 (3)	3.2 (1)
>43.94, ≤52.66 nmol/L	19.7 (24)	22.3 (29)	29.7 (22)	12.5 (7)	14.0 (12)	12.7 (7)	16.1 (5)
>52.66 nmol/L	20.5 (25)	20.8 (27)	20.3 (15)	21.4 (12)	76.7 (66)	74.6 (41)	80.7 (25)

^a^ Values are median and interquartile range (IQR) and (%) *n*. ^b^ Note: No values of serum folate were <6.8 nmol/L; the cut-offs presented for serum folate are quintiles based on the distribution of serum folate concentrations at mid-gestation.

**Table 3:** Associations between maternal vitamin B_12_ and folate status with infant serum vitamin B_12_ concentrations.

			Univariate ^b^		Multivariate ^c^		Multivariate ^d^	
Maternal Variables	Time-Point	*n*	β (SE)	*p*-Value	β (SE)	*p*-Value	β (SE)	*p*-Value
Serum vitamin B_12_, ^a^ pmol/L	Mid-gestation	54	0.29 (0.17)	0.09	0.28 (0.16)	0.08	0.31 (0.16)	0.06
	Delivery (All)	64	0.85 (0.12)	<0.0001	0.74 (0.12)	<0.0001	0.77 (0.12)	<0.001
	Delivery (Recruited at mid-gestation)	33	0.57 (0.20)	0.004	0.53 (0.18)	0.003	0.53 (0.16)	0.001
	Delivery (Recruited at delivery)	31	1.09 (0.13)	<0.0001	0.97 (0.14)	<0.0001	1.02 (0.12)	<0.001
<148.0 pmol/L	Mid-gestation	54	n/a	n/a	n/a	n/a	n/a	n/a
	Delivery (All)	64	−0.65 (0.16)	<0.0001	−0.54 (0.14)	0.0002	−0.62 (0.15)	<0.0001
	Delivery (Recruited at mid-gestation)	33	−0.63 (0.22)	0.004	−0.60 (0.19)	0.002	−0.56 (0.18)	0.002
	Delivery (Recruited at delivery)	31	−0.72 (0.22)	0.001	−0.59 (0.22)	0.008	−0.67 (0.21)	0.002
<221.0 pmol/L	Mid-gestation	54	−0.16 (0.15)	0.28	−0.18 (0.14)	0.20	−0.18 (0.14)	0.21
	Delivery (All)	64	−0.42 (0.12)	0.0007	−0.30 (0.12)	0.01	−0.33 (0.12)	0.008
	Delivery (Recruited at mid-gestation)	33	−0.23 (0.17)	0.17	−0.19 (0.16)	0.22	−0.26 (0.15)	0.07
	Delivery (Recruited at delivery)	31	−0.61 (0.17)	0.0004	−0.44 (0.17)	0.01	−0.41 (0.19)	0.03
Serum folate ^a^, nmol/L	Mid-gestation	53	−0.24 (0.14)	0.09	−0.24 (0.14)	0.09	−0.28 (0.15)	0.06
	Delivery (All)	64	0.12 (0.16)	0.47	0.07 (0.16)	0.68	0.09 (0.17)	0.61
	Delivery (Recruited at mid-gestation)	33	0.09 (0.22)	0.69	0.12 (0.23)	0.60	0.16 (0.26)	0.54
	Delivery (Recruited at delivery)	31	0.12 (0.25)	0.63	0.06 (0.23)	0.81	0.11 (0.22)	0.62
<40.0 nmol/L	Mid-gestation	53	0.10 (0.12)	0.40	0.11 (0.12)	0.37	0.14 (0.13)	0.28
	Delivery (All)	64	−0.06 (0.13)	0.65	0.02 (0.13)	0.87	0.02 (0.13)	0.88
	Delivery (Recruited at mid-gestation)	33	−0.05 (0.17)	0.76	−0.08 (0.17)	0.64	0.04 (0.20)	0.86
	Delivery (Recruited at delivery)	31	−0.08 (0.21)	0.69	0.11 (0.18)	0.56	0.15 (0.17)	0.39

^a^ Statistical analyses: Linear regression models were used to examine associations between maternal vitamin B_12_ and folate status and infant serum vitamin B_12_ concentrations; vitamin B_12_ and folate concentrations were natural logarithmically transformed prior to analyses; ^b^ Adjusted for gestational age of sample collection; ^c^ Adjusted for gestational age of sample collection, maternal age at delivery, parity (≥1 vs. 0), ever smoked (yes vs. no), relationship status (single vs. married/in a relationship), self-reported prenatal supplement use (≥2 vs. <2 pills/week), pre-pregnancy BMI, and race (African American vs. other); ^d^ Adjusted for gestational age of sample collection, maternal age at delivery, parity (≥1 vs. 0), ever smoked (yes vs. no), relationship status (single vs. married/in a relationship), self-reported prenatal supplement use (≥2 vs. <2 pills/week), pre-pregnancy BMI, race (African American vs. other), intake of vitamin B_12_, and intake of folate.

**Table 4:** Associations between maternal Vitamin B_12_ and folate status with infant serum folate concentrations.

			Univariate ^b^		Multivariate ^c^		Multivariate ^d^	
Maternal Variables	Time-Point	*n*	β (SE)	*p*-Value	β (SE)	*p*-Value	β (SE)	*p*-Value
Serum vitamin B_12_,^a^ pmol/L	Mid-gestation	51	−0.04 (0.16)	0.79	−0.19 (0.14)	0.17	−0.16 (0.13)	0.22
	Delivery (All)	61	−0.02 (0.11)	0.88	−0.08 (0.11)	0.48	−0.08 (0.11)	0.45
	Delivery (Recruited at mid-gestation)	30	−0.20 (0.15)	0.18	−0.20 (0.13)	0.13	−0.22 (0.12)	0.07
	Delivery (Recruited at delivery)	31	0.14 (0.16)	0.37	0.04 (0.15)	0.78	0.06 (0.15)	0.67
<148.0 pmol/L	Mid-gestation	51	n/a	n/a	n/a	n/a	n/a	n/a
	Delivery (All)	61	0.07 (0.13)	0.60	0.16 (0.12)	0.16	0.18 (0.12)	0.14
	Delivery (Recruited at mid-gestation)	30	0.26 (0.17)	0.13	0.26 (0.14)	0.07	0.38 (0.13)	0.005
	Delivery (Recruited at delivery)	31	−0.10 (0.18)	0.57	0.07 (0.17)	0.65	0.03 (0.17)	0.88
<221.0 pmol/L	Mid-gestation	51	−0.05 (0.14)	0.71	0.01 (0.13)	0.91	0.07 (0.12)	0.54
	Delivery (All)	61	−0.01 (0.09)	0.90	0.05 (0.09)	0.58	0.07 (0.09)	0.47
	Delivery (Recruited at mid-gestation)	30	0.18 (0.12)	0.12	0.22 (0.11)	0.04	0.27 (0.10)	0.006
	Delivery (Recruited at delivery)	31	−0.19 (0.13)	0.15	−0.09 (0.12)	0.45	−0.12 (0.13)	0.37
Serum folate ^a^, nmol/L	Mid-gestation	50	0.27 (0.14)	0.06	0.05 (0.13)	0.69	0.003 (0.13)	0.98
	Delivery (All)	61	0.54 (0.09)	<0.0001	0.47 (0.10)	<0.0001	0.50 (0.10)	<0.0001
	Delivery (Recruited at mid-gestation)	30	0.54 (0.11)	<0.0001	0.55 (0.13)	<0.001	0.53 (0.15)	0.0003
	Delivery (Recruited at delivery)	31	0.57 (0.13)	<0.0001	0.45 (0.13)	0.0005	0.44 (0.12)	0.0003
<40.0 nmol/L	Mid-gestation	50	−0.25 (0.12)	0.03	−0.13 (0.10)	0.21	−0.09 (0.10)	0.39
	Delivery (All)	61	−0.42 (0.08)	<0.0001	−0.40 (0.08)	<0.0001	−0.42 (0.08)	<0.0001
	Delivery (Recruited at mid-gestation)	30	−0.44 (0.09)	<0.0001	−0.43 (0.10)	<0.0001	−0.51 (0.12)	<0.0001
	Delivery (Recruited at delivery)	31	−0.41 (0.12)	0.0006	−0.32 (0.12)	0.01	−0.33 (0.12)	0.006

^a^ Statistical analyses: linear regression models were used to examine associations between maternal vitamin B_12_ and folate status and infant serum vitamin B_12_ concentrations; vitamin B_12_ and folate concentrations were natural logarithmically transformed prior to analyses; ^b^ Adjusted for gestational age of sample collection; ^c^ Adjusted for gestational age of sample collection, maternal age at delivery, parity (≥1 vs. 0), ever smoked (yes vs. no), relationship status (single vs. married/in a relationship), self-reported prenatal supplement use (≥2 vs. <2 pills/week), pre-pregnancy BMI, and race (African American vs. other); ^d^ Adjusted for gestational age of sample collection, maternal age at delivery, parity (≥1 vs. 0), ever smoked (yes vs. no), relationship status (single vs. married/in a relationship), self-reported prenatal supplement use (≥2 vs. <2 pills/week), pre-pregnancy BMI, race (African American vs. other), intake of vitamin B_12_, and intake of folate
